# The CSR-1 endogenous RNAi pathway ensures accurate transcriptional reprogramming during the oocyte-to-embryo transition in *Caenorhabditis elegans*

**DOI:** 10.1371/journal.pgen.1007252

**Published:** 2018-03-26

**Authors:** Christina Fassnacht, Cristina Tocchini, Pooja Kumari, Dimos Gaidatzis, Michael B. Stadler, Rafal Ciosk

**Affiliations:** 1 Friedrich Miescher Institute for Biomedical Research, Basel, Switzerland; 2 University of Basel, Basel, Switzerland; 3 Swiss Institute of Bioinformatics, Basel, Switzerland; 4 Institute of Bioorganic Chemistry, Polish Academy of Sciences, Poznan, Poland; University of California San Diego, UNITED STATES

## Abstract

Endogenous RNAi (endoRNAi) is a conserved mechanism for fine-tuning gene expression. In the nematode *Caenorhabditis elegans*, several endoRNAi pathways are required for the successful development of reproductive cells. The CSR-1 endoRNAi pathway promotes germ cell development, primarily by facilitating the expression of germline genes. In this study, we report a novel function for the CSR-1 pathway in preventing premature activation of embryonic transcription in the developing oocytes, which is accompanied by a general Pol II activation. This CSR-1 function requires its RNase activity, suggesting that, by controlling the levels of maternal mRNAs, CSR-1-dependent endoRNAi contributes to an orderly reprogramming of transcription during the oocyte-to-embryo transition.

## Author summary

During the oocyte-to-embryo transition, the control of development is transferred from the mother to the embryo. A key event during this transition is the transcriptional activation of the embryonic genome, which is tightly controlled. Here, by using the nematode *C*. *elegans*, we uncover a role for endogenous RNA interference in this process. We demonstrate that a specific endoRNAi pathway, employing the Argonaute protein CSR-1, functions as a break on gene-specific, and potentially global, activation of embryonic transcription in the developing oocytes. Our findings reveal a new layer of control over the transcriptional reprogramming during the oocyte-to-embryo transition, raising questions about its potential conservation in mammalian development.

## Introduction

Developmental plasticity, or pluripotency, is acquired during the oocyte-to-embryo transition, during which a highly specialized cell, the oocyte, is reprogrammed into a developmentally flexible embryo [1]. A tight regulation of this reprogramming is critical, since germ cells that precociously acquire pluripotency can give rise to teratomas, which are germ cell tumors characterized by the presence of differentiated somatic cells. Like in mammals, teratomas in *C*. *elegans* can arise in the absence of reprogramming-controlling RNA binding proteins (RBPs) [2]. The *C*. *elegans* RBPs GLD-1/Quaking and LIN-41/TRIM71 inhibit a precocious reprogramming of germ cells into somatic-like cells at consecutive stages of oogenic development [3, 4]. Similar to the natural order of events during the oocyte-to-embryo transition, somatic-like differentiation of *gld-1* and *lin-41* germ cells is preceded by reactivation of the cell cycle and embryonic genome activation (EGA) [3–5]. During earlier stages of oogenic development, inhibition of the cell cycle by GLD-1 is critical to prevent precocious reprogramming [5]. The reprogramming-related function of LIN-41 in differentiating oocytes remains less clear, though it may also involve cell cycle regulation [4, 6, 7]. To better understand the control of reprogramming in the oocytes, we performed an unbiased genetic screen, searching for mutants displaying precocious activation of embryonic transcription in the oocyte-containing (proximal) part of the gonad, and identified components of endogenous small interfering RNA (endo-siRNA) pathways.

There are two main endo-siRNA pathways operating in the *C*. *elegans* germline, which are named after the constituent Argonaute proteins; the WAGO (*worm argonaute*) and the CSR-1 (*chromosome-segregation and RNAi-deficient-1*) pathway [8, 9]. These Argonautes bind specific classes of small RNAs, which are 22 nucleotides in length with a guanosine at the 5’end (22G RNAs) [8]. 22G RNAs are produced by RNA-dependent RNA polymerases (RdRPs) and act as secondary effectors of the endo-siRNA pathways. CSR-1 is the only worm Argonaute protein required for fertility and embryo survival [10]. It has been reported to function in diverse processes, including chromosome segregation [9, 10], chromatin organization [9, 11], histone mRNA processing [12], germ granule formation [9, 13], alternative splicing [14], and exogenous RNAi [10]. CSR-1 binds small RNAs that are complementary to most germline-expressed genes [9], and has been suggested to promote the expression of these target genes [15]. Moreover, CSR-1 has been suggested to counteract gene silencing by recognizing and licensing self-sequences for expression [16–18]. Among the reported inhibitory roles of CSR-1 are the translational repression of FBF-1 target mRNAs in mitotic germ cells [19], and the degradation of germline-expressed mRNAs dependent on the RNA-slicing activity of CSR-1 [20].

In this study, we present a new role for the CSR-1 pathway and its RNA-slicing activity in ensuring the transcriptional silencing of embryonic genes in developing oocytes. In wild-type development, the oocyte-to-embryo transition takes place in the absence of Pol II-dependent transcription. In slicer-inactive *csr-1* mutants, however, the global Pol II inhibition is compromised. Our observations suggest a model, where CSR-1–dependent titration of maternal mRNAs is required to delay the onset of embryonic transcription until its physiological onset in early embryos.

## Results

### Identification of new EGA inhibitors

**Fig 1:**
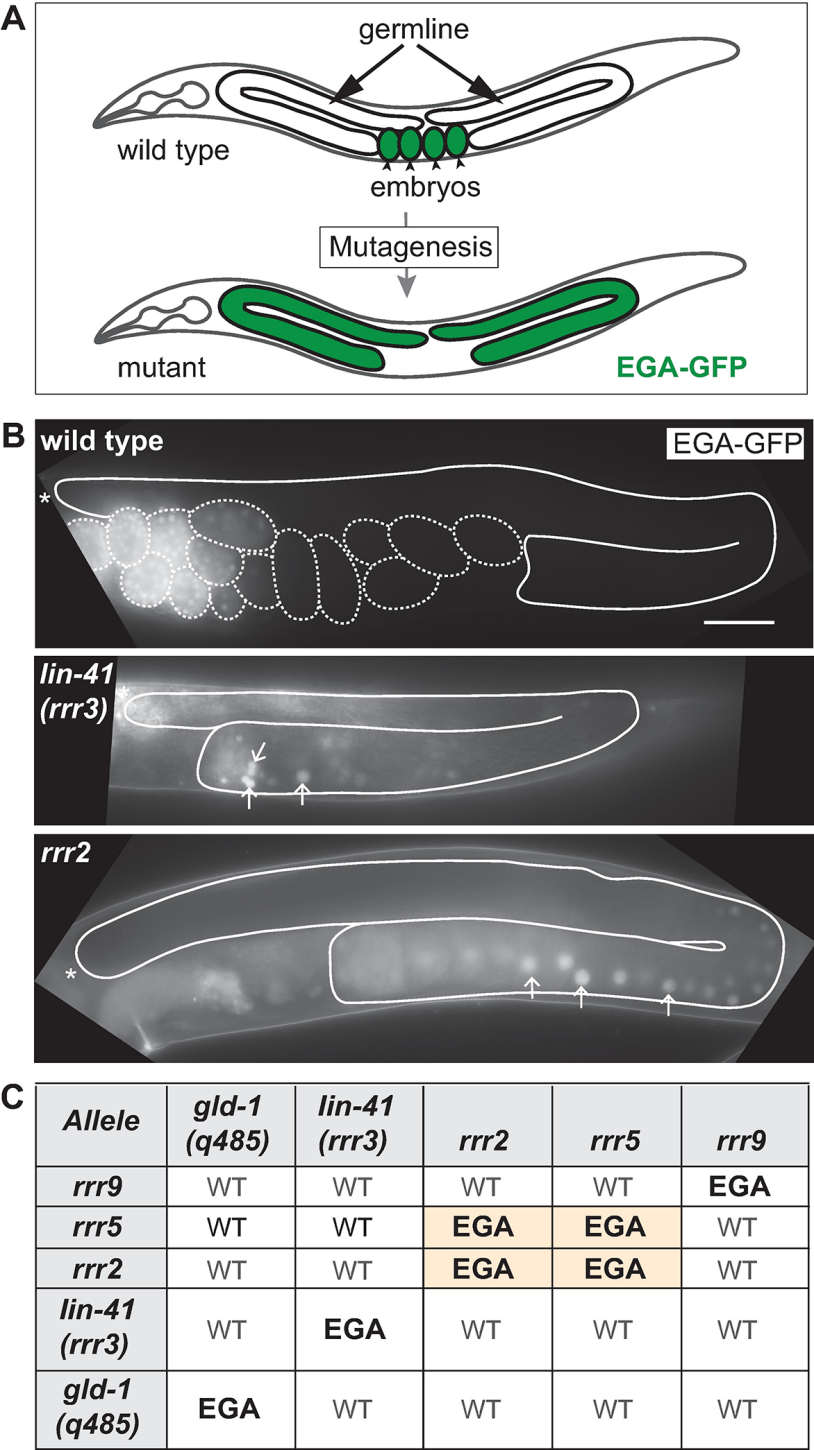
Identification of new EGA repressors. (A) Schematic representation of a genetic screen, performed to identify novel regulators of EGA in the adult germline. The EGA-GFP reporter was used to monitor transcriptional reprogramming occurring during the oocyte-to-embryo transition (green indicates embryonic transcription). Wild-type animals express EGA-GFP in embryos. Mutant animals abnormally express this reporter in germ cells. (B) Fluorescent micrographs of live animals expressing the EGA-GFP reporter. Here, and in the subsequent figures, gonads are outlined with a continuous line and asterisks mark the distal end of the gonad. The dashed line in the top panel outlines the embryos. While wild-type animals express the EGA-GFP specifically in embryos, the *lin-41(rrr3)* mutants express it in proximal gonadal cells undergoing a germline-to-soma transition. The new mutants (the *rrr2* mutant is shown as an example) express EGA-GFP precociously in developing oocytes. Arrows point to three representative, arbitrary chosen, nuclei displaying EGA-GFP. Scale bar: 40 μm. (C) Complementation matrix of newly (*rrr2*, *rrr5*, and *rrr9*) and previously (*gld-1(q485)* and *lin-41(rrr3)*) identified mutants displaying premature EGA. “WT” indicates complementation and “EGA” non-complementation. The *rrr2* and *rrr5* mutants did not complement each other, suggesting that they affect the same gene.

During the oocyte-to-embryo transition, the RBPs GLD-1 and LIN-41 inhibit the onset of embryonic transcription in oogenic germ cells [4, 5]. The EGA-inhibiting function of LIN-41 was uncovered through a genetic screen, in which a premature onset of embryonic transcription in the adult germline was monitored by GFP, expressed from the promoter of a gene expressed as soon as EGA takes place, *vet-4* (henceforth the EGA-GFP reporter) [4]. Here, we continued this approach to identify additional EGA inhibitors (Fig 1A). We isolated three independent mutants (*rrr2*, *rrr5* and *rrr9*) expressing the EGA-GFP reporter precociously in germ cells (Fig 1B). Complementation tests, using these mutants alongside *gld-1* and *lin-41*, suggested the identification of two new genes; one affected by the *rrr2* and *rrr5* mutations, and the other by the *rrr9* mutation (Fig 1C).

### In the isolated mutants, EGA is uncoupled from cell cycle defects

**Fig 2:**
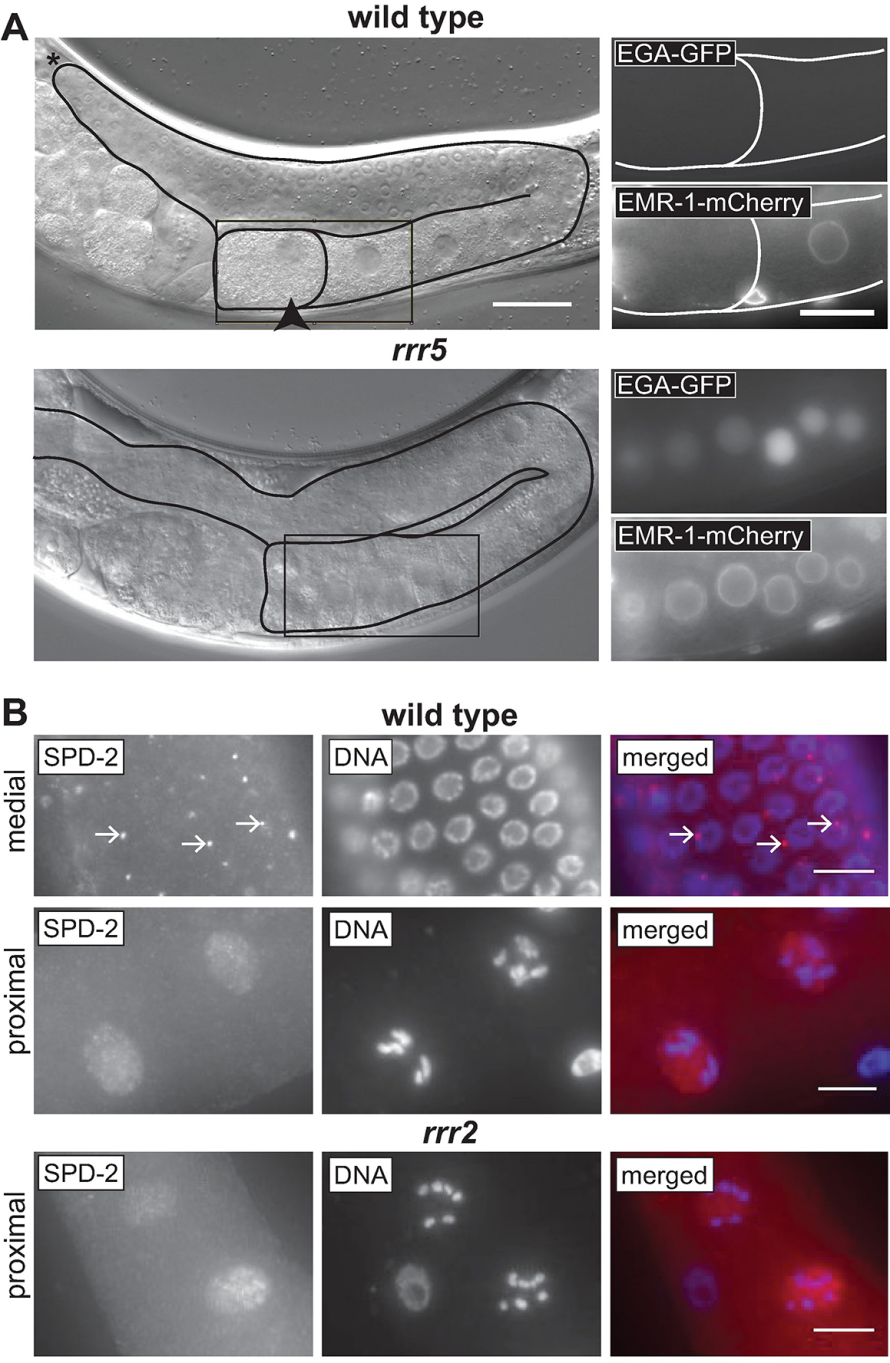
Precocious EGA is uncoupled from cell cycle progression. (A) DIC micrographs of live animals, either wild-type or *rrr5*. Boxed areas are enlarged on the right, showing EGA-GFP or EMR-1:mCherry to visualize EGA or nuclear envelope breakdown, respectively. Scale bars: 30 μm (left) and 20 μm (right). In wild type, the oocytes display EMR-1 within nuclear envelopes, except for the ovulating -1 oocyte (outlined), which underwent nuclear envelope breakdown. The *rrr5* oocytes, which express EGA-GFP, have EMR-1–decorated intact nuclear envelopes, suggesting that the premature EGA is independent from ovulation/cell cycle progression. (B) Fluorescent micrographs of wild-type and *rrr2* germlines from the indicated gonadal regions, immunostained for the centrosomal component SPD-2 and stained with DAPI. Scale bars: 10 μm. In the medial gonad, SPD-2 is present within centrosomes (examples indicated by the arrows); this staining is absent from both wild-type and *rrr2* oocytes in the proximal gonad, indicating normal centrosome elimination.

The abnormal EGA observed in *gld-1* or *lin-41* germ cells occurs in the context of a premature exit from meiosis and the onset of mitotic proliferation, which is followed by teratoma formation [3, 4, 6, 7, 21]. In addition, EGA can be induced by precocious oocyte maturation [22]. Oocyte maturation occurs in wild-type gonads in the (-1) oocyte adjacent to the spermatheca, and is characterized by nuclear envelope breakdown (NEBD), cytoskeletal rearrangements and meiotic spindle assembly [23]. To test whether EGA in the new mutants reflects precocious oocyte maturation, we examined the NEBD in these mutants. To visualize nuclear envelopes, we used a strain expressing mCherry-tagged nuclear envelope protein, EMR-1 [24]. We observed that the new mutants expressed EGA-GFP in the absence of NEBD (Fig 2A), indicating that EGA is not induced by precocious oocyte maturation. To follow it further, we examined additional events accompanying the progression through meiosis. In wild-type, but not in *gld-1* or *lin-41* mutants, centrosomes are eliminated during oocyte differentiation [7, 25, 26]. We monitored the centrosomes in *rrr2* mutants by staining the centrosomal protein SPD-2 [27], and observed the loss of centrosomes in mutant oocytes (Fig 2B). Thus, similar to wild type, the mutants appear to eliminate centrosomes from the oocytes. Finally, wild-type oocytes, which arrest in the diakinesis stage of meiosis I, display highly condensed bivalent chromosomes. In the mutants, we observed similarly condensed chromosomes (Fig 2B), suggesting a relatively normal meiotic progression and arrest. Thus, in contrast to the previously reported cases of precocious EGA, in the new mutants EGA appears to be uncoupled from reactivation of the cell cycle.

### EGA, but not embryonic-like differentiation, is induced in the mutants

**Fig 3:**
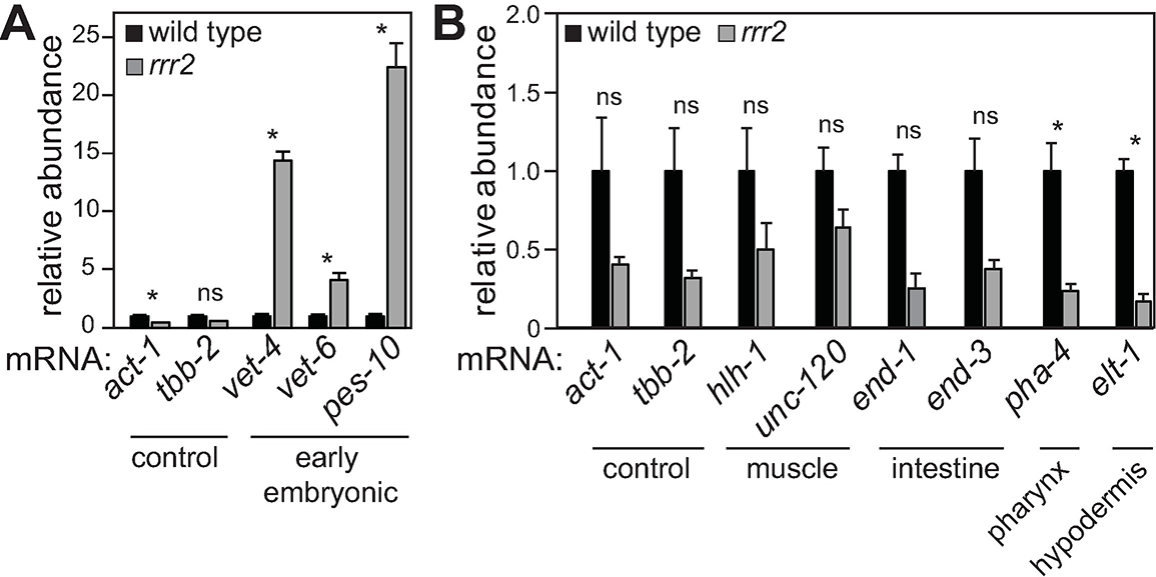
EGA, but not embryonic-like differentiation, is induced in the mutants. (A) Examination of EGA transcripts, by RT-qPCR, in gonads of the indicated genotypes. “Control”: ubiquitous mRNAs. “Early embryonic”: mRNAs expressed immediately after the onset of EGA. Each bar represents the mean of three independent biological replicates. Error bars represent the SEM. The significance of the differences has been calculated with the Student’s t-test: “*”, p<0.05; “*n*.*s*.”, not significant. (B) Detection of somatic lineage-specific transcripts, by RT-qPCR, in gonads of the indicated genotypes. “Muscle”, “intestine”, etc, indicate somatic lineages characterized by expression of the indicated mRNAs. Each bar represents the mean of three independent biological replicates. Error bars and significance were calculated as in A.

To characterize further embryonic transcription in the mutant gonads, we examined expression of the endogenous *vet-4* and several additional embryonic transcripts, using reverse transcription and quantitative PCR (RT-qPCR). We found that the tested early embryonic transcripts (*vet-4*, *vet-6* and *pes-10*) were all upregulated in the gonads dissected from *rrr2* mutants, compared to wild-type gonads (Fig 3A). In *gld-1* or *lin-41* gonads, precocious EGA is followed by embryonic-like differentiation resulting in teratomas [4, 5]. To examine whether *rrr2* germ cells attempt somatic differentiation, we examined the expression of several transcripts specific to somatic lineages. We found that these transcripts were not upregulated in the dissected *rrr2* gonads (Fig 3B). Thus, the *rrr2* mutant germ cells appear to execute the first step in the transcriptional oocyte-to-embryo reprogramming, but in contrast to *gld-1* or *lin-41* mutants, do not undergo further somatic-like differentiation.

**Fig 4:**
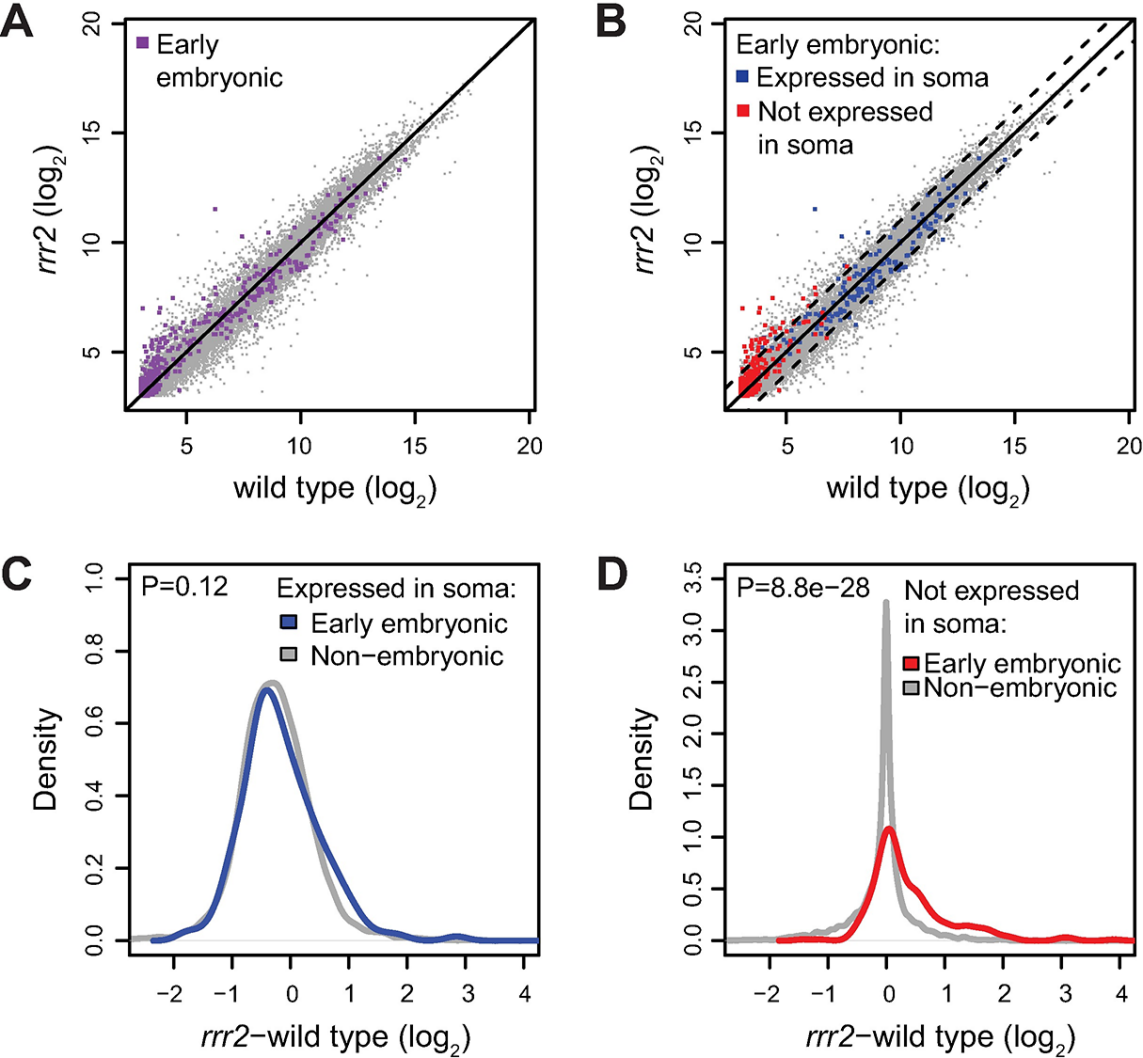
Embryonic genes are globally induced in mutant animals. (A) Scatter plot comparing transcript abundance in wild type (x-axis) and *rrr2* animals (y-axis). Colored in purple: Early embryonic transcripts (selected as described in Materials and Methods). (B) The plot as in A. Colored in blue: early embryonic transcripts *expressed* in the soma. Colored in red: early embryonic transcripts *not* expressed in the soma. Somatic expression was determined from RNA-seq in *glp-4* mutants (Material and Methods). Dashed lines indicate 2-fold changes. (C) Density plots depicting expression changes in *rrr2*, compared to wild type, for early embryonic (blue) and non-embryonic (grey) transcripts *expressed* in the soma. No difference between the two populations of transcripts could be detected (P = 0.12, p-value calculated with 2-sample Wilcoxon test). (D) Same as C, but considering only transcripts *not* expressed in the soma, with early embryonic transcripts colored in red. In this case, the population of early embryonic transcripts showed a positive shift (P = 8.8 x 10^−28^, p-value calculated with 2-sample Wilcoxon test), indicating their upregulation in the mutant.

To examine the extent of embryonic transcription in the *rrr2* animals, we performed RNA sequencing on wild type and *rrr2* mutants (expression changes between the two biological replicates are shown in S1 Fig and S1 Dataset). We selected 446 early embryonic transcripts based on the transcriptome profiling of staged embryos [28] (see Material and Methods). Some of these transcripts were expressed in the wild type (Fig 4A). Because we used young adults prior to embryo production, we suspected that this reflects their additional expression in the adult soma. Thus, any changes in these transcripts in the germline would be likely masked by their somatic expression. To circumvent this problem, we split the embryonic transcripts between somatically expressed and not expressed (based on the previous analysis of germline-specific expression [29]) (Fig 4B). Consistently, the embryonic transcripts, which were also expressed in the soma, were not upregulated in the *rrr2* mutant compared to wild type (p-value: 0.12; calculated with 2-sample Wilcoxon test, Fig 4C). In contrast, the “strictly” embryonic genes, which were not somatically expressed in the wild type, showed a mild, but significant upregulation in the *rrr2* mutant (p-value: 8.8 x 10^−28^; Fig 4D). These results are consistent with a widespread de-repression of embryonic genes in the mutant gonads.

### Precocious EGA in the mutants reflects defects in the CSR-1 endoRNAi pathway

**Fig 5:**
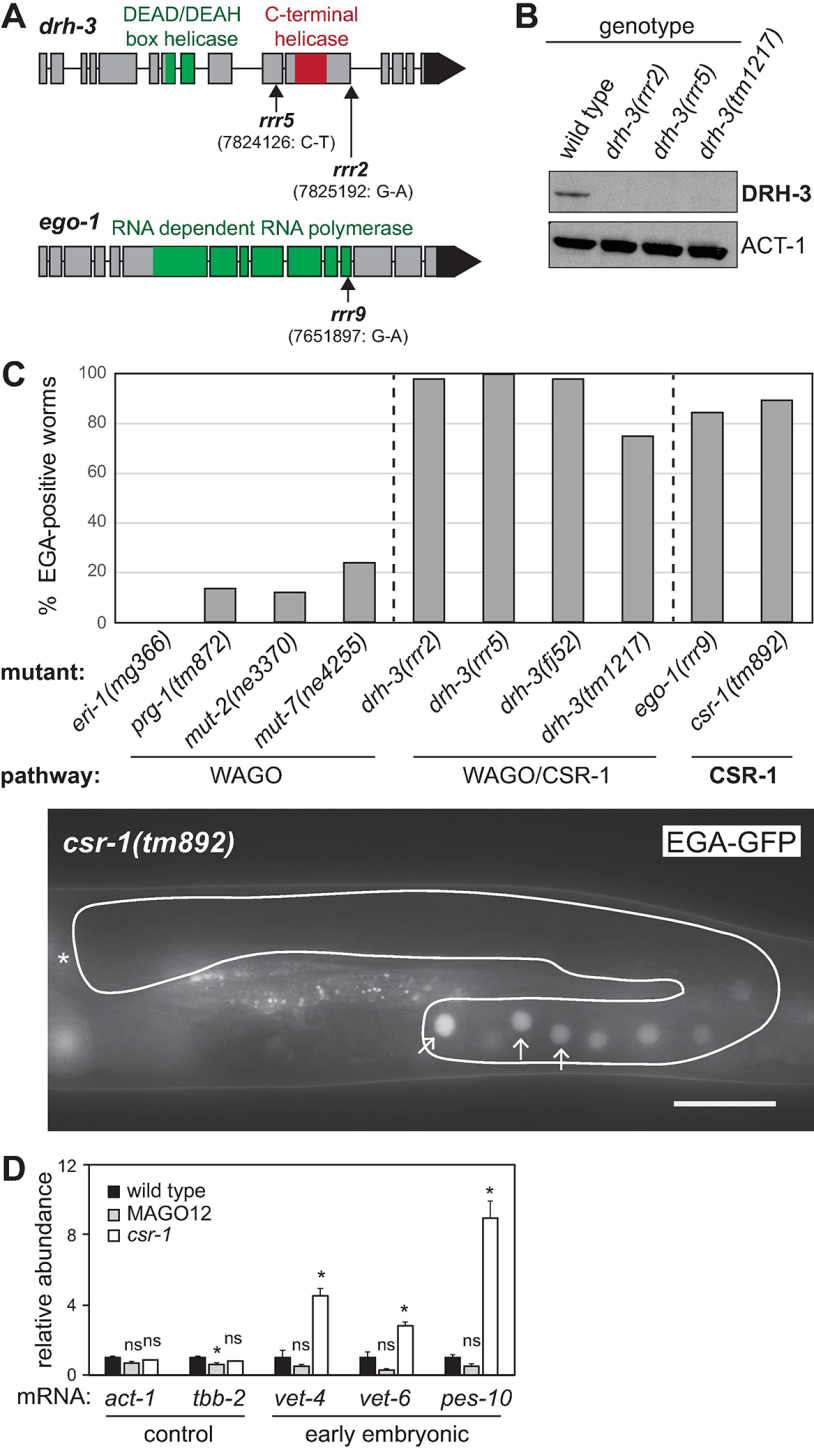
EGA repression in the germline is mediated *via* the CSR-1 pathway. (A) Schematic view of the *drh-3* and *ego-1* genes with highlighted known protein domains. Arrows indicate the locations of the identified mutations. The *drh-3(rrr2)* mutation affects a splice donor residue, likely leading to a splicing defect. The *drh-3(rrr5)* mutation, located in exon 10, is causing a precocious stop codon (Q647>Stop). The *ego-1(rrr9)* mutation is located in exon 12, and leads to a nonsynonymous amino acid change (A1271>V). Genomic locations according to *C*. *elegans* May 2008 (WS190/ce6) assembly. (B) Western blot analysis of lysates from wild type or *drh-3* mutants, probed for DRH-3 and ACT-1 (as a loading control). No DRH-3 protein was detected in the lysates from *drh-3(rrr2)* and *drh-3(rrr5)* animals. The *drh-3(tm1217)* mutation, which was used as a control, is a putative null (containing a 482 bp deletion). (C) Top: Examination of EGA-GFP in mutants affecting various small RNA pathways. Each bar represents the percentage of animals expressing the reporter in germ cells. N>30. Bottom: Fluorescent micrograph from a live *csr-1(tm892)* animal, grown at 25°C, expressing the EGA-GFP abnormally in the developing oocytes (arrows point to three representative oocytes). Scale bar: 40 μm. (D) Changes of the indicated transcripts in gonads dissected from wild type, a strain containing mutations in all 12 WAGOs (MAGO12), and *csr-1(tm892)* mutants, by RT-qPCR. Each bar represents the mean of three independent biological replicates. Error bars represent the SEM. The significance of the differences has been calculated with the Student’s t-test: “*”, p<0.05; “*n*.*s*.”, not significant.

We mapped the mutants to *drh-3* (*dicer-related-helicase-3*, alleles *rrr2* and *rrr5*) and to *ego-1* (*enhancer-of-GLP-ONE-1*, allele *rrr9*) (Fig 5A and S2 Fig); both functioning in small non-coding RNAi pathways. All mutants behaved like molecular nulls, displaying fully penetrant sterility as reported earlier for the reference *drh-3* and *ego-1* alleles [30, 31]. Consistently, DRH-3 protein was not detectable in *drh-3(rrr2 or rrr5)* mutants by western blot (Fig 5B). DRH-3 is a core component of all RdRP complexes and therefore important for the biogenesis of all classes of 22G-RNAs [8]. EGO-1 functions either redundantly with another RdRP, called RRF-1, in the production of WAGO 22G-RNAs [8], or alone in the production of CSR-1 22G RNAs [9]. To test which of the two pathways controls EGA, we examined expression of the EGA-GFP reporter in mutants affecting both or just one of the two pathways. In *drh-3* mutants (both newly isolated and reference) that affect both pathways, we observed the expected gonadal EGA-GFP (Fig 5C). Different primary siRNA-pathways, functioning upstream of the WAGO pathway, use the RdRP complex for the production of secondary 22G RNAs to enhance their effects. They include the maternal ERGO-1 and spermatogenesis-specific ALG-3/4 26G RNA pathways [32–34], as well as the piRNA pathway [35–37]. To address if precocious EGA results from disrupting the 26G RNA pathways, we examined the expression of EGA-GFP in *eri-1(mg366)* mutants lacking the 26G RNAs [33]. To test the involvement of the piRNA pathway, we examined *prg-1(tm892)* mutants lacking most piRNAs [38]. We found that neither *eri-1* nor *prg-1* mutants expressed EGA-GFP in the gonads (Fig 5C). Likewise, *mut-2* or *mut-7* mutants, which are deficient in the amplification of WAGO 22G RNAs [8, 39, 40], displayed only occasional gonadal EGA-GFP. In contrast, 90% of the *csr-1(tm892)* mutants displayed the gonadal expression of EGA-GFP, which was comparable to *ego-1(rrr9)* or *drh-3* mutants (Fig 5C). To verify that the observed gonadal EGA-GFP reflects the expression of endogenous embryonic genes, we examined several embryonic transcripts by RT-qPCR. Consistent with the expression of the EGA-GFP reporter, we observed increased expression of embryonic genes in the gonads from *csr-1(tm892)* mutants, but not the gonads from the MAGO12 strain, which carries mutations in all WAGO genes [8] (Fig 5D). Together, these results suggest that it is the CSR-1 pathway that prevents precocious EGA.

### The CSR-1 slicer activity is required for EGA inhibition

**Fig 6:**
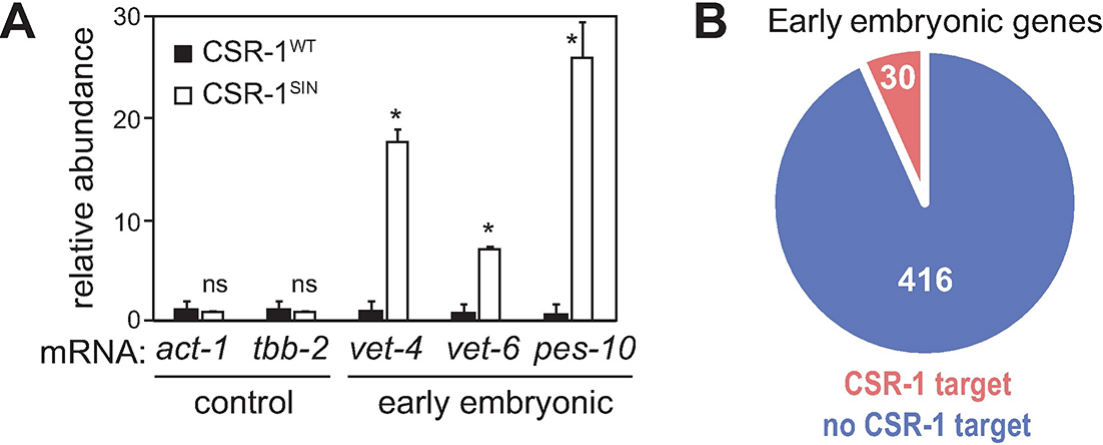
The slicing activity of CSR-1 is required to inhibit EGA. (A) Detection of EGA transcripts by RT-qPCR in gonads from animals of the indicated genotypes. The *csr-1(tm892)* animals were expressing either wild-type CSR-1 (CSR-1^WT^), or a CSR-1 variant with mutations compromising its slicing activity (CSR-1^SIN^); only the gonads from the latter strain expressed embryonic genes. Each bar represents the mean of three independent biological replicates. Error bars and p-values indicated as explained before. (B) Among a group of 446 early-expressed embryonic genes, only 30 are potentially targeted by CSR-1, based on the presence of antisense 22G RNAs associated with CSR-1.

Recently, CSR-1 has been reported to contribute to maternal mRNA regulation *via* its endonuclease activity (also RNase or slicer activity) [20]. To test whether the slicer activity is required to prevent precocious EGA, we examined, by RT-qPCR, the expression of several embryonic transcripts in gonads dissected from *csr-1(tm892)* animals, expressing either rescuing FLAG-tagged CSR-1 [18] or a FLAG-tagged CSR-1 variant, in which the DDH catalytic slicer residues were mutated to AAA (obtained from Craig Mello’s lab). Similar to other *csr-1* loss-of-function mutations, the *s*licer-*in*active CSR-1 (CSR-1^SIN^) protein was unable to prevent the gonadal expression of embryonic transcripts (Fig 6A). Thus, the RNase activity of CSR-1 is required to prevent precocious EGA.

CSR-1 is thought to contribute to normal embryonic development by regulating the abundance of maternal transcripts loaded into the embryo [20]. Thus, to address whether CSR-1 may directly target early embryonic transcripts, we examined whether these transcripts have complementary CSR-1 22G RNAs. Early embryonic transcripts were determined as described before (see Material and Methods). We then defined the number of putative CSR-1 targets based on the presence of complementary 22G RNAs associated with CSR-1 [9]. This analysis showed that a great majority of early embryonic transcripts (416/446 genes) did not have any complementary CSR-1 22G RNAs (Fig 6B); among the embryonic transcripts that we routinely tested by RT-qPCR, only *vet-6* had complementary 22G RNAs. Moreover, a variant of the EGA-GFP reporter, in which the *vet-4* 3’UTR was replaced by a non-regulated *tbb-2* 3’UTR, thus limiting the regulation of this reporter to the *vet-4* promoter, was also de-repressed in CSR-1–depleted gonads (S3 Fig). Combined, these observations suggest that CSR-1 is unlikely to regulate EGA by directly degrading embryonic transcripts.

### The gonadal EGA correlates with defective Pol II inhibition

**Fig 7:**
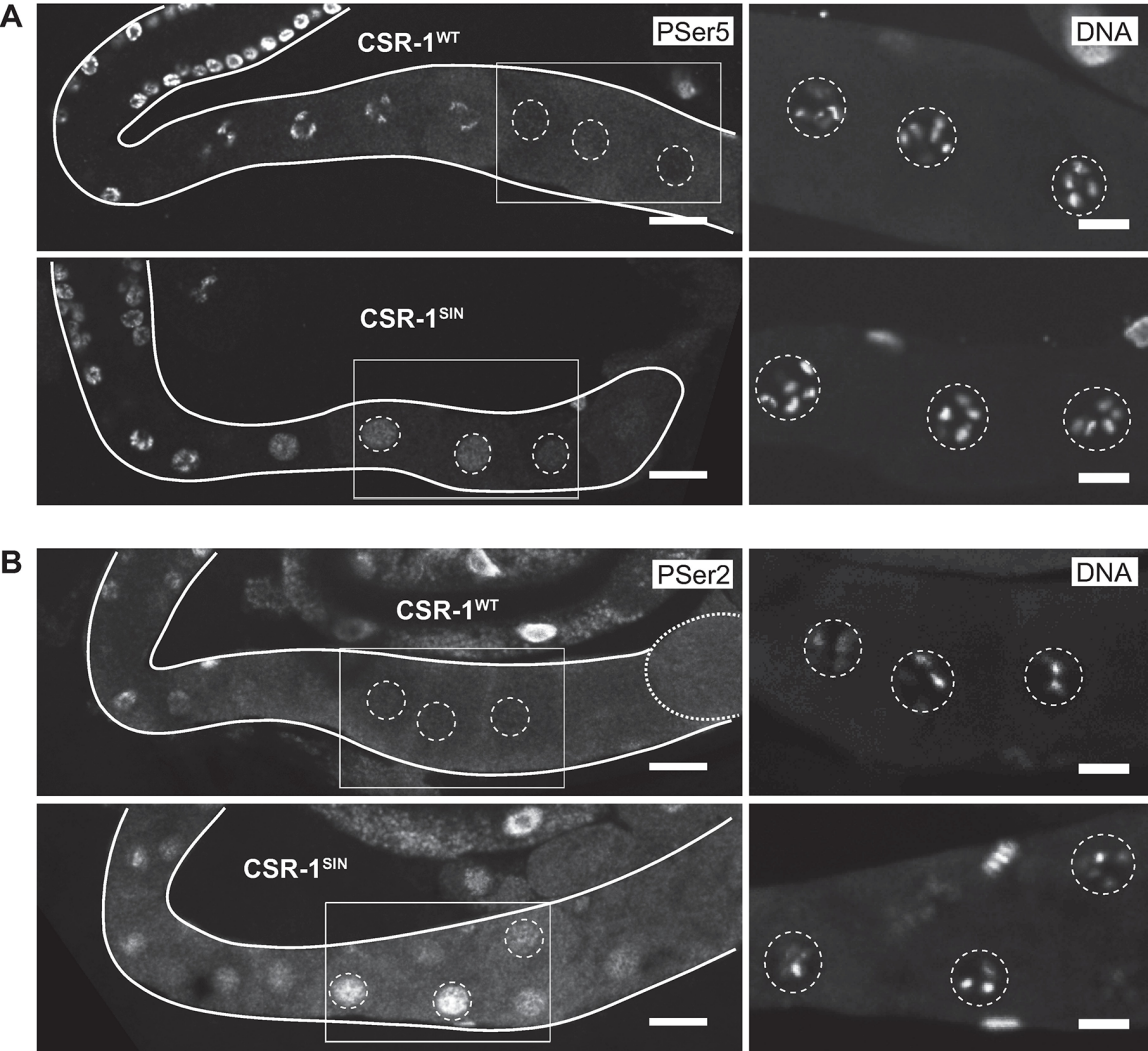
The slicing activity of CSR-1 is required for Pol II inhibition. (A) Fluorescent confocal micrographs showing gonads from *csr-1(tm892)* animals expressing either CSR-1^WT^ or CSR-1^SIN^. On the left, the gonads were immunostained for phosphorylated serine 5 (PSer5) within the C-terminal domain of RNA-Polymerase II, indicating transcriptional initiation. Notice the oocyte staining in CSR-1^SIN^ (but not CSR-1^WT^) -expressing gonads. Scale bars: 20 μm. The boxed areas are magnified on the right, showing the same gonads stained for DNA by DAPI, to visualize the highly compacted chromosomes characteristic of arrested oocytes. The dotted lines encircle oocyte nuclei. Scale bars: 10 μm. (B) Median intensity projections of confocal micrographs showing gonads immunostained for the serine 2 phosphorylation of Pol II CTD (PSer2), which indicates transcriptional elongation. Notice the oocyte staining in the CSR-1^SIN^ (but not CSR-1^WT^) -expressing gonad. Scale bars: 20 μm. The boxed areas are magnified on the right, as in A. Scale bars: 10 μm.

A global repression of Pol II-dependent transcription is a hallmark of the oocyte-to-embryo transition. In *C*. *elegans*, Pol II transcription is silenced from late stages of meiosis I in oocytes until the onset of EGA in early embryos [41–44]. The inhibition of Pol II is manifested by the loss of activating phosphorylations on Pol II C-terminal domain (CTD); serine 5 is phosphorylated (PSer5) during transcription initiation and serine 2 (PSer2) during the elongation [44]. To explore whether the premature EGA observed in the slicer-inactive *csr-1* mutants might reflect defective Pol II inhibition, we stained the gonads of CSR-1^SIN^-expressing animals [20] with antibodies specific to PSer5 and PSer2. We found that, in contrast to animals expressing CSR-1^WT^_,_ the oocytes in CSR-1^SIN^-expressing animals contained Pol II phosphorylated at both Ser5 and Ser2 (Fig 7A and 7B); these phosphoepitope-specific stainings corresponded to Pol II, as they were diminished in CSR-1^SIN^ animals depleted of the Pol II large subunit AMA-1 (S4 Fig). These findings suggest that, in the absence of CSR-1-dependent slicing, the oocytes no longer undergo Pol II inhibition, potentially linking it to the premature expression of embryonic genes.

## Discussion

CSR-1 has been recently shown to function as an RNase that, by degrading certain maternal transcripts, ensures proper embryonic development [20]. Although it remains possible that the point mutations in CSR-1 affecting its RNase activity might compromise interactions with other factors, our findings suggest that CSR-1 uses its RNA-slicing activity to control embryonic event(s) already in the oocytes, where it prevents a precocious activation of embryonic transcription. The regulation by CSR-1 is expected to involve 22G RNAs, which are associated with CSR-1 to guide it to its mRNA targets. Thus, the observation that most early embryonic transcripts lack complementary 22G RNAs implies that CSR-1 is unlikely to control EGA by directly degrading EGA transcripts.

In one scenario, CSR-1 could control embryonic transcription indirectly by degrading mRNA encoding a transcriptional regulator. In other species, EGA depends on specific transcription factors, which recognize sequence motifs in the embryonic promoters [45]. However, an analogous transcription factor has not been found in *C*. *elegans* to date. Thus, we tested whether the promoters of early embryonic genes misregulated in *drh-3* animals are enriched for the binding motifs of specific transcription factors. Although we observed enrichment for the binding motifs of HMG-12 and LIN-29, these motifs are very abundant in the promoters of all embryonic genes (S5 Fig), potentially leading to a bias in the statistical analysis. Moreover, previous analysis argued against LIN-29 function in the activation of embryonic genes [4].

In embryos, EGA is controlled by a mechanism relying on the inhibition of general transcription factors. In one- and two-cell stage embryos, TAF-4, a TFIID subunit, is sequestered in the cytoplasm by the RNA-binding proteins OMA-1/2, thereby preventing the nuclear function of TAF-4 in Pol II-dependent transcription [46]. In four-cell stage embryos, the degradation of OMA-1/2 permits the nuclear translocation of TAF-4, consequently allowing TFIID assembly, Pol II transcription, and EGA. Our observation that Pol II remains activated in slicer-inactive *csr-1* oocytes suggests a possible connection between CSR-1 and Pol II repression. While the levels of most mRNAs encoding Pol II subunits appeared to remain constant in the absence of CSR-1 slicing activity, the levels of *taf-11*.*2* (encoding a general transcription factor) and *cit-1*.*2* (a subunit of the transcription elongation factor pTEFb) were increased [20]. Thus, CSR-1–mediated degradation of the corresponding mRNAs could potentially explain the link between CSR-1, Pol II regulation, and EGA. However, depleting CSR-1 from *taf-11*.*2* or *cit-1*.*2* mutants failed to suppress germline EGA (our observation), suggesting that the potential degradation of these targets by CSR-1 is not critical to inhibit EGA.

In another indirect scenario, CSR-1 could control EGA by ensuring wild-type chromatin architecture, which could involve either cytoplasmic or nuclear functions of CSR-1. For example, CSR-1 has been implicated in the biogenesis of histone mRNAs [12]. However, reducing histone levels (by depleting CDL-1, another histone mRNA biogenesis factor) did not result in oocyte expression of the EGA reporter (S6 Fig). Other effects on chromatin organization resulting from the depletion of CSR-1 pathway components include mislocalization of the centromere-specific histone HCP-3/CENP-A [9, 15, 47] and the loss of H3K9me2 deposition on unpaired chromatin [11]. Whether the CSR-1 slicer function is required for these chromatin-related functions of CSR-1 is not yet clear. Thus, we cannot exclude the possibility that the premature EGA and abnormal activation of Pol II are indirectly linked to altered chromatin.

Summarizing, our studies reveal an unexpected role for the CSR-1 endoRNAi pathway in inhibiting the expression of embryonic genes during oocyte development. While the majority of existing studies suggested a positive function for CSR-1 in promoting germline expression, this and the recent study by Gerson-Gurwitz et al. suggest a negative function, involving its RNA-slicer activity. Whether CSR-1 controls EGA by degrading mRNA encoding a specific transcriptional regulator, by affecting chromatin, or through an entirely different mechanism, remains an open question. Another open issue is the relation of CSR-1 to the previously identified EGA repressors, particularly LIN-41, which, like CSR-1, represses EGA in the developing oocytes [4]. In *lin-41* mutants, premature EGA and subsequent teratomatous differentiation are coupled to the re-activation of the cell cycle [4, 5, 22]. By contrast, premature EGA in CSR-1 pathway mutants is independent from the cell cycle. Because *lin-41* mutants display several oocyte-to-embryo transition events, but *csr-1* mutants only EGA, CSR-1 might function “downstream” from LIN-41. Importantly, also mammalian oocytes express and utilize endo-siRNAs [48]. These endo-siRNAs are produced by an oocyte-specific Dicer isoform, Dicer^O^, which lacks the N-terminal DExD helicase domain; the loss of Dicer^O^ from oocytes results in meiotic arrest, with spindle and chromosome segregation defects, resulting in sterility [49]. The deletion of AGO2, the only mammalian Argonaute with slicer activity, results in a similar oocyte phenotype, and the slicer activity of AGO2 is essential for the oocyte function of endo-siRNAs [50]. Thus, the regulation by endo-siRNAs is a conserved feature of oocyte biology, and the potential role of mammalian endo-siRNAs in controlling the embryonic genome remains an exciting possibility.

## Methods

### Nematode culture, mutants and transgenic lines

Animals were maintained using standard procedures and were grown at 20°C unless stated otherwise. For alleles and transgenic lines used in this study, see the S1 Table.

### Mutagenesis and whole-genome-sequencing (WGS)

The screen was performed as previously described [4]. The *rrr2* mutant was identified using strain #1284, the EGA-GFP reporter strain, as a parent strain for mutagenesis. The *rrr5* and *rrr9* mutants were discovered using strain #1270 as parent strain, which contains in addition to the EGA-GFP reporter a transgene to visualize P granules and a *glo-1* mutation to reduce autofluorescence from the gut. Mapping of the mutants was performed as described before [4]. Before WGS, each mutant was outcrossed 4–8 times to the unmutagenized parent strain. Genomic DNA was isolated using Gentra Puregene Tissue Kit (Qiagen). DNA libraries were made from 50 ng of genomic DNA using the Nextera DNA kit from Illumina. Sequencing was performed using HiSeq 2000 from Illumina.

### Processing of sequence data and detection of sequence variants

The analysis was performed similarly as described in [4]. Briefly, the sequence reads were aligned to the May 2008 *C*. *elegans* assembly (obtained from http://hgdownload.soe.ucsc.edu/goldenPath/ce6/chromosomes/) using ‘‘bwa” [51]; version 0.7.4) with default parameters, but only retaining single-hit alignments (‘‘bwa samse -n 1” for single reads and “bwa sampe -a 1000 -o 1000 -n 1” for paired end reads and selecting alignments with ‘‘X0:i:1”). The resulting alignments were converted to BAM format, sorted and indexed using ‘‘samtools”[52]; version 0.1.19). In order to quantify contamination by *Escherichia coli*, reads were similarly aligned to a collection of *E*. *coli* genomes (NCBI accession numbers NC_008253, NC_008563, NC_010468, NC_004431, NC_009801, NC_009800, NC_002655, NC_002695, NC_010498, NC_007946, NC_010473, NC_000913 and AC_000091), which typically resulted in less than 1% aligned reads. Potential PCR duplicates were identified and removed using Picard (version 1.92, http://broadinstitute.github.io/picard/), reducing the number of reads to 27% to 44% in single read samples, and to 93% in the paired-end read sample. Sequence variants were identified using GATK [53] (version 2.5.2) following recommended “best practice variant detection”: initial alignments were first corrected by indel realignment and base quality score recalibration, followed by SNP and INDEL discovery and genotyping using “UnifiedGenotyper” for each individual strain using standard hard filtering parameters, resulting in a total of five to six thousand sequence variations in each strain compared to the reference genome. Finally, the number of high quality (score > = 500) single nucleotide substitutions of EMS-type (G/C>A/T transitions [54]) not found in the parent strain (typically less than 1% of the total number of variants per strain) were counted in sequential windows of 1 Mb to identify regions of increased variant density.

### Mutant mapping

We performed complementation crosses between the newly identified mutants, and mutants affecting the previously identified EGA repressors *gld-1* and *lin-41* (using the *gld-1(q485)* and *lin-41(rrr3)* alleles). Due to sterility of the homozygous mutants, we crossed heterozygous mutants and examined the F1 progeny for the precocious EGA/sterility phenotype occurring with expected penetrance. Only in the crosses between heterozygous *rrr2* and *rrr5* mutants did we observe non-complementation in the F1 progeny, suggesting that the mutations affected the same gene. Based on the whole-genome sequencing (see above), the only gene displaying sequence alterations in both mutants was *drh-3*. We confirmed the *drh-3* mutations as causal, based on non-complementation with the published *drh-3(fj52)* mutant, and by observing precocious EGA upon *drh-3* RNAi and in other *drh-3* mutants (alleles *fj52* and *tm1217*). The *rrr9* mutant contained only few variants of the EMS-type; among those only four variants were predicted to affect the corresponding proteins by non-synonymous substitutions. Among the candidates, only *ego-1* mutants were previously reported to be sterile. The mutation in e*go-1* was confirmed to be causal, based on non-complementation with the published *ego-1(om71)* mutant and a precocious EGA was observed upon *ego-1* RNAi.

### Reverse transcription and quantitative PCR on dissected gonads

RNA was isolated from 50 gonads of 1-day-old (after the L4-to-adult molt) animals using the Picopure RNA Isolation Kit (Arcturus). Three independent biological replicates were collected. cDNAs were generated using the QuantiTect Reverse Transcription Kit (Qiagen). Real-time PCR was performed in duplicates (for each biological replicate) using Absolute QPCR SYBR green ROX mix (AbGene) on an ABI PRISM 7700 system or a StepOnePlus system (both Applied Biosystems). qPCR reactions were performed as described previously [5]. At least one primer in each pair was specific for an exon-exon junction (see primer sequences in the S2 Table). Standard curves for every primer pair were generated using a serial dilution of cDNA from embryos and were used to determine the amount of each transcript in the gonads. All technical duplicate values were first averaged, and then values for a particular transcript from tested animals were normalized to the mean value for the corresponding transcript from control animals. The normalized means of the samples were used to make the bar plots. Error bars show the standard error of the mean (SEM) for the three biological replicates. We used *act-1*, encoding an actin isoform, and *tbb-2*, encoding a tubulin isoform, as reference transcripts as they are ubiquitously expressed housekeeping genes. Statistical analysis of the data was performed using two-sided Student’s t-test, assuming non-equal variance, in R. Each comparison was performed separately without correcting for multiple statistical tests.

### RNA sequencing and data analysis

For total RNA sequencing, we collected samples from young adult *drh-3(rrr2)* mutants and wild type (N2) animals in duplicates. The RNA sequencing samples from wild type have been uploaded previously to the GEO database (series GSE62858, GSM1534604 and GSM1534605) [29]. RNA extraction and library preparation were performed as described before [29]. The samples were sequenced on an Illumina HiSeq 2500. The total RNA sequencing data was analyzed as previously described [55]. Differential gene expression analysis for *rrr2* was performed using the edgeR package from Bioconductor [56], and is presented in the S1 Dataset.

### Selection of genes activated in the early embryo

We downloaded microarray expression data [28] for the samples (GSM39513-GSM39519, GSM39543, GSM39522-GSM39526, GSM39530 and GSM39531) from GEO (www.ncbi.nlm.nih.gov/geo/), representing the 4-cell (3x), 8-cell (4x), 15-cell (4x) and 26-cell (4x) stages. The data was normalized using the function justGCRMA from the Bioconductor package gcrma. We confirmed a high degree of consistency among the replicates and then averaged those to obtain one expression profile per stage. Embryonic transcription in *C*. *elegans* starts at the 4-cell stage [57]. Therefore, early embryonic genes were defined as genes that showed a change in expression of at least 2-fold between the 4-cell and the 8-cell stage, or between the 8-cell and the 15-cell stage and had no expression at the 4-cell stage (expression < 3.75). Maternally provided genes were defined as genes that showed expression at the 4-cell stage (expression > = 3.75). Genes not falling in any of the two above categories were defined as non-embryonic. Data from the 26-cell stage were not used for selecting genes, as there was only little change in expression when comparing to the 15-cell stage. To identify the somatically expressed early embryonic transcripts, we used RNA sequencing data (GSE62857) from dissected gonads and germline-less *glp-4* animals [29]. We processed and normalized the data as described previously [29] and defined somatic transcripts using a cutoff of 5 (log2).

### EGA reporter assay

Temperature-sensitive mutants used for the EGA reporter assay in Fig 5C were grown at 15°C. Mutant animals were synchronized by bleaching and put as L1 larvae on OP50 plates to 25°C until scored for EGA-GFP expression as adults.

### Immunostaining

Immunostaining against SPD-2 (“969LA”, 1:800) was performed as previously described [4]. A Zeiss Axio Imager Z1 microscope equipped with an Axiocam MRm REV 2 CCD camera was used for capturing pictures. Immunostainings against PSer5 of Pol II CTD (“3E8”, Millipore, 1:25) and PSer2 of Pol II CTD (“3E10”, Millipore, 1:25) were performed as previously described [44]. DNA was visualized using 4-6-Diamidino-2-phenylindole (DAPI). Pictures were taken using an Axio Imager M2 microscope and a Yokogawa CSU W1 Duel camera. Images were processed with Fiji and Adobe Photoshop in an identical manner and imported into Adobe Illustrator.

### CSR-1 slicer inactive mutants

For RT-qPCR analysis (Fig 6A), we used strain 1689 for CSR-1^WT^ [18], and strain 1755 for CSR-1^SIN^ (kindly shared by the Mello group prior to publication). The Pol II CTD staining experiments (Fig 7A and 7B) were performed using published strains 1905 for CSR-1^WT^ and 1900 for CSR-1^SIN^ [20].

### Protein extraction and western blot analysis

Synchronized adult worms were harvested for protein extraction as described before [5]. The *drh-3* homozygous populations were obtained by worm sorting. Proteins were resolved by SDS-PAGE on 4–12% Bis-Tris Protein Gels (NuPAGE Novex) and transferred using the Trans-Blot Turbo Transfer System (BIO RAD). Membranes were blocked with 4% milk in PBST and incubated with the primary antibodies α-DRH-3 [58] or α-ACT-1 (MAB1501, Millipore) in blocking buffer at 4°C overnight. Membranes were washed three times in PBST and incubated with the secondary (HRP-conjugated) antibody (GE Heathcare) in blocking buffer at RT for 1 hour, and washed again three times with PBST. Chemiluminescence was performed using Pierce ECL Western Blotting Substrate (Thermo Scientific).

## Supporting information

S1 Fig Expression changes RNA-sequencing data. Scatter plot depicting expression changes in *drh-3(rrr2)*, compared to wild type, for the two biological replicates. r = Correlation coefficient.
(PDF)

S2 Fig Single-nucleotide variants in the isolated mutants. After EMS-mutagenesis, all mutants were outcrossed against the parental strain 5 to 8 times. “chr” indicate chromosomes and “M” mitochondrial DNA. Numbers of detected single nucleotide variants (SNVs) are indicated on the y-axis. The detected variants were filtered, retaining only high quality single nucleotide variants (quality score of at least 500) of EMS-type (G/C -> A/T transitions) that were not found in the parent strain.
(PDF)

S3 Fig Premature EGA is determined by the promoter, rather than the 3’UTR. Fluorescent micrographs of live animals expressing the *Pvet-4*::*mCherry*:*h2b*::*tbb-2 3’UTR* reporter. Control RNAi-treated animals did not express this reporter in germ cells, whereas *csr-1* RNAi-treated animals expressed it in the oocytes. Arrows point to three representative, arbitrary chosen, nuclei displaying reporter expression. Scale bar: 40 μm.
(PDF)

S4 Fig PSer5 and PSer2 epitopes in CSR-1^SIN^ oocyte nuclei are Pol II-dependent. (A) Median intensity projections of confocal micrographs showing gonads from *csr-1(tm892)* animals expressing CSR-1^SIN^. In *control* (empty vector) RNAi-treated animals phosphorylated serine 5 (PSer5) is present, whereas in *ama-1* RNAi-treated animals PSer5 in diakinetic oocyte nuclei is absent. Scale bars: 20 μm. The boxed areas are magnified on the right, showing the same gonads stained for DNA by DAPI, to visualize the compacted chromosomes characteristic of arrested oocytes. The dotted lines encircle oocyte nuclei. Scale bars: 10 μm. (B) Same as in A, but gonads are immunostained for serine 2 phosphorylation (PSer2). The PSer2 staining detected in diakinetic oocyte nuclei of *control* RNAi-treated animals is abolished upon *ama-1* RNAi treatment. Scale bars: 20 μm. The boxed areas are magnified on the right, as in A. Scale bars: 10 μm.
(PDF)

S5 Fig Transcription factor binding site analysis. Analysis was performed using the “not expressed in soma early embryonic genes” (see Fig 4). X-axis: overall abundance of the binding sites for each transcription factor; Y-axis: directed p-value calculated by t-test (positive values indicate enrichment, negative values indicate depletion in *drh-3(rrr2)*, as compared to wild type). A significant hit is expected to have a directed p-value of greater than 3.64 (see Supporting Methods, indicated by the gray horizontal line). Each circle represents one transcription factor. The two candidates above the line are HMG-12 and LIN-29.
(PDF)

S6 Fig CDL-1 depletion does not cause EGA reporter expression in germ cells. Fluorescent micrographs of live animals expressing the EGA-GFP reporter following control or *cdl-1* RNAi treatment. *Cdl-1* RNAi-treated animals were sterile, producing only unfertilized oocytes. Like control RNAi treated animals, also *cdl-1* RNAi treated animals did not show EGA-GFP expression in germ cells (1 out of 45 sterile animals). Scale bar: 30 μm.
(PDF)

S1 Dataset Expression changes RNA-seq data. (XLSX)

S1 Table The *C*. *elegans* strains used in this study. (PDF)

S2 Table Real-time quantitative PCR primer sequences. (PDF)

S1 Text Supporting methods. (PDF)
