# Supplementary figures and images for: The CSR-1 endogenous RNAi pathway ensures accurate transcriptional reprogramming during the oocyte-to-embryo transition in Caenorhabditis elegans
Source: PLoS Genet. 2018 Mar 26;14(3):e1007252. doi: 10.1371/journal.pgen.1007252 (PMC5886687; doi:10.1371/journal.pgen.1007252)

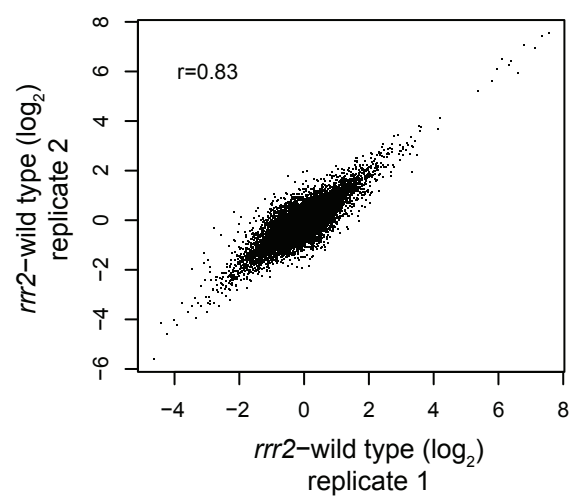

Supplement: S1 Fig — Scatter plot depicting expression changes in drh-3(rrr2), compared to wild type, for the two biological replicates. r = Correlation coefficient. (PDF) [file pgen.1007252.s001.pdf]

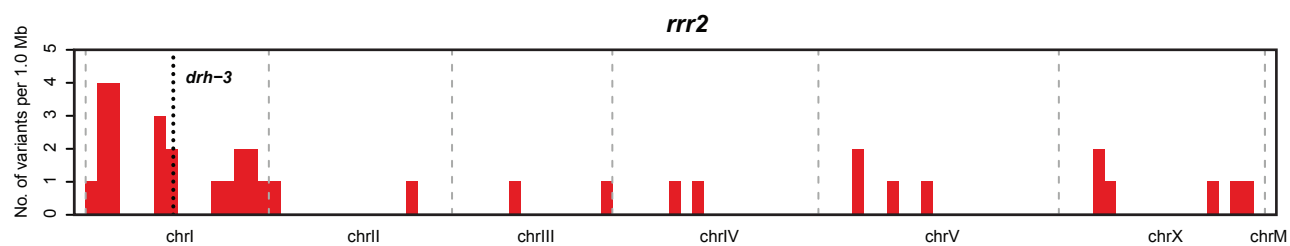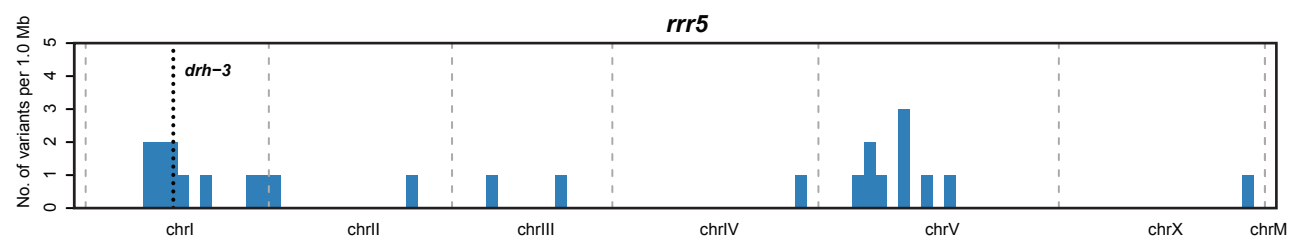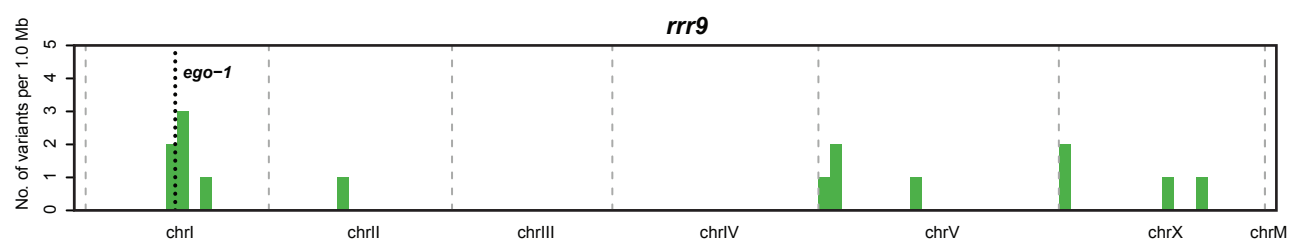

Supplement: S2 Fig — After EMS-mutagenesis, all mutants were outcrossed against the parental strain 5 to 8 times. “chr” indicate chromosomes and “M” mitochondrial DNA. Numbers of detected single nucleotide variants (SNVs) are indicated on the y-axis. The detected variants were filtered, retaining only high quality single nucleotide variants (quality score of at least 500) of EMS-type (G/C -> A/T transitions) that were not found in the parent strain. (PDF) [file pgen.1007252.s002.pdf]

Pvet-4::mCherry:h2b::tbb-2 3'UTR

control RNAi

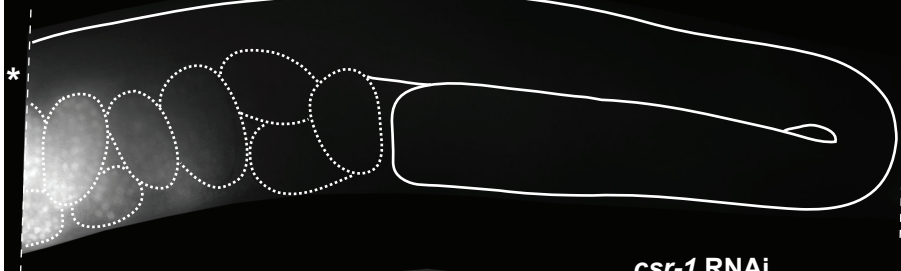

csr-1 RNAi

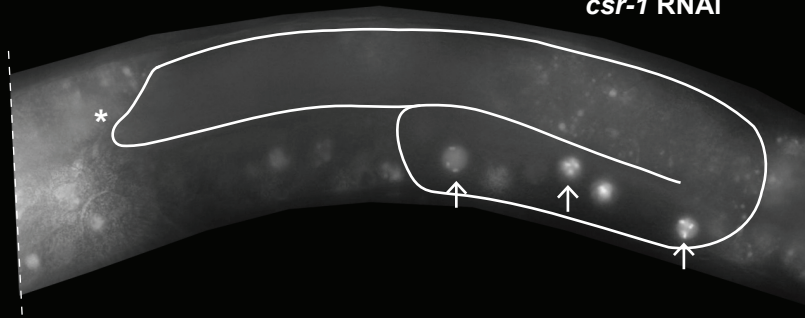

Supplement: S3 Fig — Fluorescent micrographs of live animals expressing the Pvet-4::mCherry:h2b::tbb-2 3’UTR reporter. Control RNAi-treated animals did not express this reporter in germ cells, whereas csr-1 RNAi-treated animals expressed it in the oocytes. Arrows point to three representative, arbitrary chosen, nuclei displaying reporter expression. Scale bar: 40 μm. (PDF) [file pgen.1007252.s003.pdf]

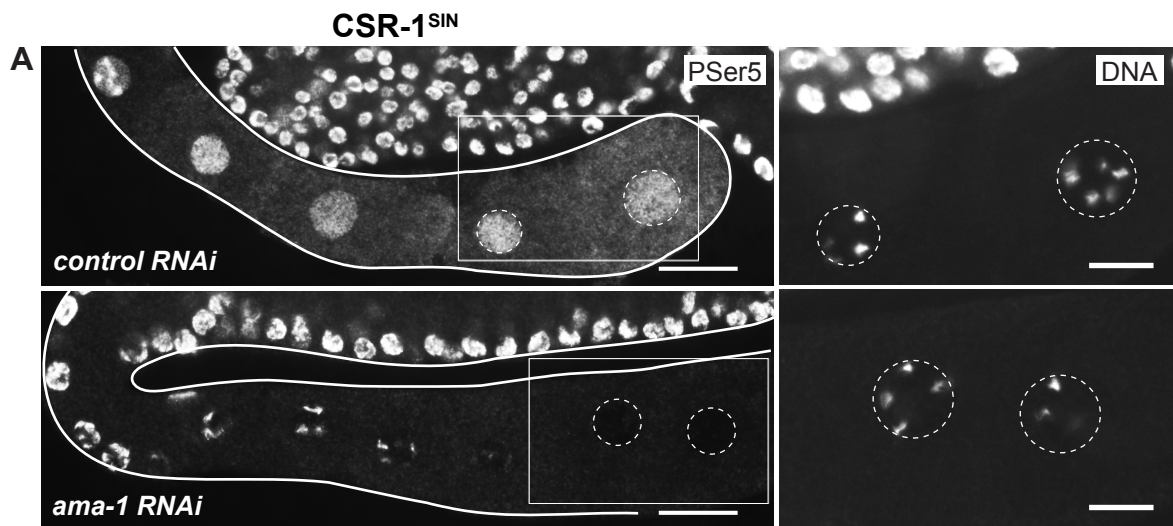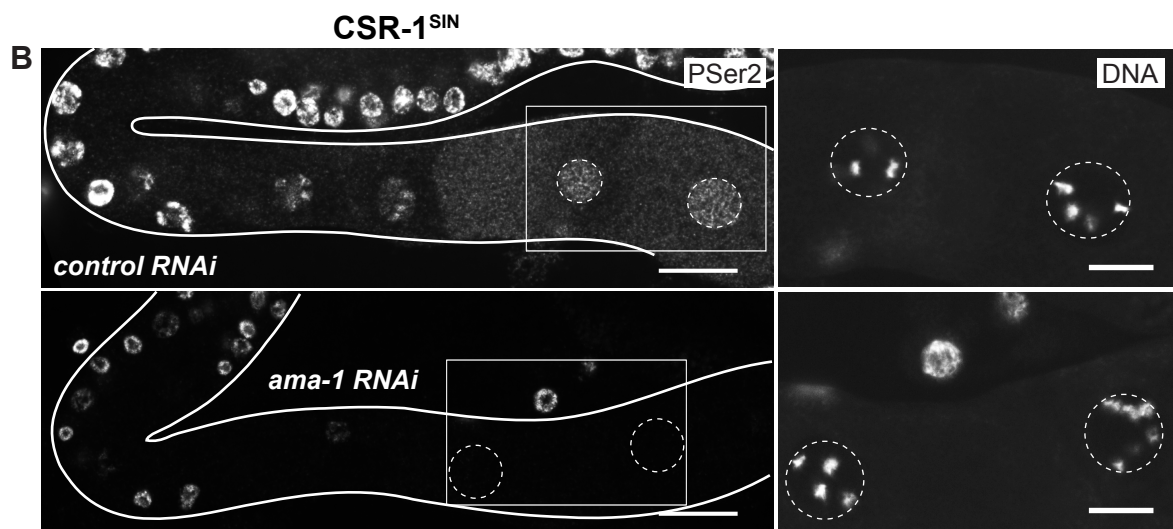

Supplement: S4 Fig — (A) Median intensity projections of confocal micrographs showing gonads from csr-1(tm892) animals expressing CSR-1SIN. In control (empty vector) RNAi-treated animals phosphorylated serine 5 (PSer5) is present, whereas in ama-1 RNAi-treated animals PSer5 in diakinetic oocyte nuclei is absent. Scale bars: 20 μm. The boxed areas are magnified on the right, showing the same gonads stained for DNA by DAPI, to visualize the compacted chromosomes characteristic of arrested oocytes. The dotted lines encircle oocyte nuclei. Scale bars: 10 μm. (B) Same as in A, but gonads are immunostained for serine 2 phosphorylation (PSer2). The PSer2 staining detected in diakinetic oocyte nuclei of control RNAi-treated animals is abolished upon ama-1 RNAi treatment. Scale bars: 20 μm. The boxed areas are magnified on the right, as in A. Scale bars: 10 μm. (PDF) [file pgen.1007252.s004.pdf]

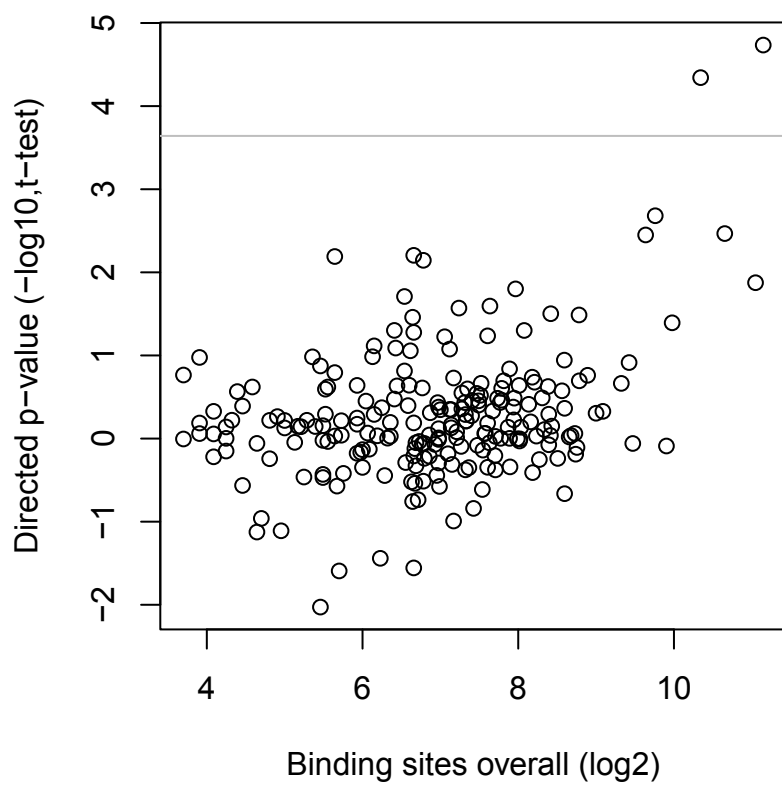

Supplement: S5 Fig — Analysis was performed using the “not expressed in soma early embryonic genes” (see Fig 4). X-axis: overall abundance of the binding sites for each transcription factor; Y-axis: directed p-value calculated by t-test (positive values indicate enrichment, negative values indicate depletion in drh-3(rrr2), as compared to wild type). A significant hit is expected to have a directed p-value of greater than 3.64 (see Supporting Methods, indicated by the gray horizontal line). Each circle represents one transcription factor. The two candidates above the line are HMG-12 and LIN-29. (PDF) [file pgen.1007252.s005.pdf]

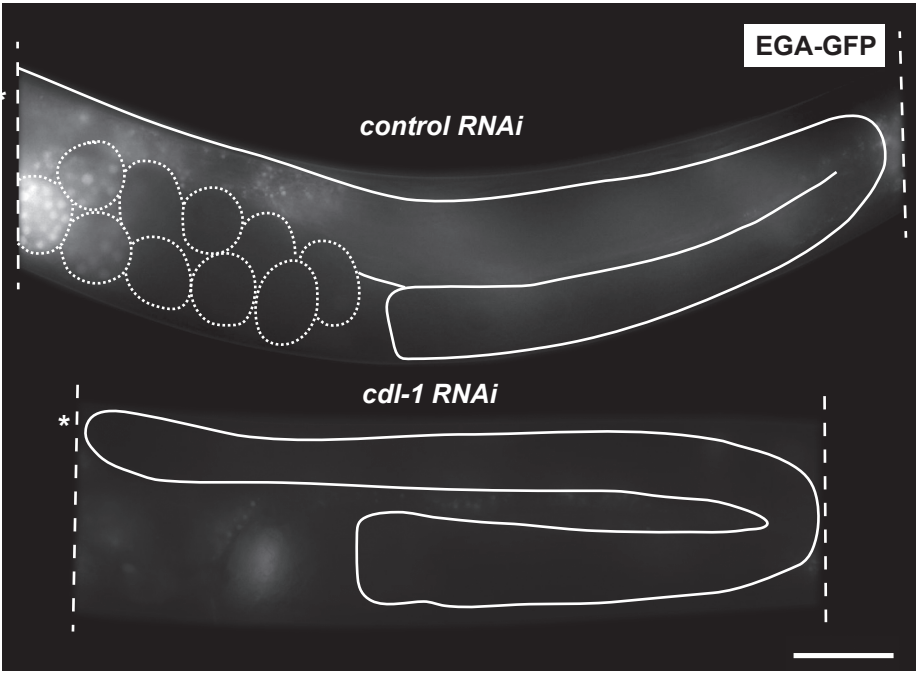

Supplement: S6 Fig — Fluorescent micrographs of live animals expressing the EGA-GFP reporter following control or cdl-1 RNAi treatment. Cdl-1 RNAi-treated animals were sterile, producing only unfertilized oocytes. Like control RNAi treated animals, also cdl-1 RNAi treated animals did not show EGA-GFP expression in germ cells (1 out of 45 sterile animals). Scale bar: 30 μm. (PDF) [file pgen.1007252.s006.pdf]
