# Supplementary material for: The CSR-1 endogenous RNAi pathway ensures accurate transcriptional reprogramming during the oocyte-to-embryo transition in Caenorhabditis elegans
Source: PLoS Genet. 2018 Mar 26;14(3):e1007252. doi: 10.1371/journal.pgen.1007252 (PMC5886687; doi:10.1371/journal.pgen.1007252)
Supplement: S1 Table — (PDF) [file pgen.1007252.s008.pdf]

S1 Table. The *C. elegans* strains used in this study

**CGC** or RAF (Ciosk lab) collection #	**Genotype**
**N2**	*Caenorhabditis elegans* wild isolate
1284	*rrrSi199[Pvet-4::NLS:gfp:gfp::vet-4 3'UTR; unc-119(+)] (II); rrrSi198[Pvet-4::NLS:gfp:gfp::vet-4 3'UTR; unc-119(+)] (IV)*
1270	*rrrSi199 (II); rrrSi198 (IV); zuIs252[nmy-2::pgl-1:RFP, unc-119(ed3)]; glo-1(zu391) (X)*
1350	*drh-3(rrr2) (I)/hT2 (I;III); rrrSi199 (II); rrrSi198 (IV); glo-1(zu391) (X)*
1431	*drh-3(rrr5) (I)/hT2 (I;III); rrrSi199 (II); rrrSi198 (IV); zuIs252; glo-1(zu391) (X)*
1600	*ego-1(rrr9) (I)/hT2 (I;III); rrrSi199 (II); rrrSi198 (IV); zuIs252; glo-1(zu391) (X)*
**JK3025**	*gld-1(q485) (I)/hT2 (I;III)*
1180	*lin-41(rrr3)/unc-29(e1072), lin-11(h1281) (I); rrrSi199 (II); rrrSi198 (IV)*
1534	*eri-1(mg366) (IV); rrrSi199 (II)*
1651	*prg-1(tm872) (I); rrrSi199 (II)*
1617	*mut-2(ne3370) (I); rrrSi199 (II)*
1616	*mut-7(ne4255) (III); rrrSi199 (II)*

1610	*drh-3(fj52) (I)/hT2 (I;III); rrrSi199 (II)*
**EL302**	*ego-1(om71) unc-29(e193) (I)/hT2 (I;III)*
1618	*avr-14(ad1302) drh-3(tm1217) (I)/hT2 (I;III) ;rrrSi199 (II)*
1596	*csr-1(tm892) (IV)/DnT1 (IV;V); rrrSi199 (II)*
**WM191**	MAGO12: *sago-2(tm894) ppw-1(tm914) ppw-2(tm110) F55A12.1(tm2686) R06C7.1(tm1414) I; Y49F6.1(tm1127) ZK1248.7(tm1113) F58G1.1(tm1019) II; C16C10.3(tm1200) K12B6.1(tm1195) III; T22H9.3(tm1186) V; R04A9.2(tm1116) X*
1646	*bqSi142[pBN20(unc-119(+) Pemr-1::emr-1:mCherry;)] (II)*
1634	*drh-3(rrr5) (I)/hT2 (I;III); bqSi142 (II); rrrSi198 (IV)*
1489	*unc-119 (III); rrrSi319[Pvet-4::mCherry:h2b::tbb-2 3'UTR; unc-119(+)] (IV)*
1689	*csr-1(tm892) (IV); Pcsr-1::FLAG:CSR-1::csr-1 3'UTR (II)*
1755	*csr-1(tm892) (IV)/DnT1 (IV;V); Pcsr-1::FLAG:CSR-1(D606A, D681A, H822A: isoform b numbering)::csr-1 3'UTR (II)*
1900	*ItSi242[pOD1267/pAG31; Pcsr-1::csr-1(reencoded; D606A, D681A: isoform b numbering), cb-unc-119(+)] II; unc-119(ed3) III?; csr-1(tm892) IV/nT1[unc-?(n754)let-?] (IV;V)*
1905	*ItSi240[pOD1265/pAG29; Pcsr-1::csr-1(reencoded), cb-unc-119(+)] II; unc-119(ed3) III?; csr-1(tm892) IV/nT1[unc-?(n754)let-?] (IV;V)*
