# Supplementary material for: The CSR-1 endogenous RNAi pathway ensures accurate transcriptional reprogramming during the oocyte-to-embryo transition in Caenorhabditis elegans
Source: PLoS Genet. 2018 Mar 26;14(3):e1007252. doi: 10.1371/journal.pgen.1007252 (PMC5886687; doi:10.1371/journal.pgen.1007252)
Supplement: S2 Table — (PDF) [file pgen.1007252.s009.pdf]

S2 Table. Real-time quantitative PCR primer sequences

Primer	Sequence
act-1 FW	CTATGTTCCAGCCATCCTTCTTGG
act-1 RV	TGATCTTGATCTTCATGGTTGATGG
tbb-2 FW	GCTCATTCTCGGTTGTACCA
tbb-2 RV	TGGTGAGGGATACAAGATGG
vet-4 FW	AAGGATTTCACTGCTTGCTC
vet-4 RV	CGTCGTTTTCGATTTCTCCG
vet-6 FW	GTGCGAGACAAGAATGTAATCC
vet-6 RV	TTCTTGAACTCTTGGAACACAG
pes-10 FW	GCGATGATTTCATGATTTCCTG
pes-10 RV	AATTTCGTAGTCAATCTGCTCC
hlh-1 FW	ACGATTATGTGACTTCCTCTC
hlh-1 RV	GATGATCTCTATCGTCGTCC
unc-120 FW	GGGTATTATGAAGAAGGCATTCG
unc-120 RV	TGCATATGTGTAGACATGACCA
end-1 FW	GGGCAATACTTTGTTCAATCG
end-1 RV	GGATACTGTTGTGAGTAGCA
end-3 FW	GCCTATTAATGACCTCCAGC

end-3 RV	CCCGTCAATTGGTATCTCTG
pha-4 FW	CCAGAATTTCCTGAACAACAC
pha-4 RV	GTTGGTGGAGCTGTAAAGAG
elt-1 FW	ACAATTCTCAATTCAGCACG
elt-1 RV	GTTGCAGAGGTAGTTTCCTG
