# Supplementary material for: The CSR-1 endogenous RNAi pathway ensures accurate transcriptional reprogramming during the oocyte-to-embryo transition in Caenorhabditis elegans
Source: PLoS Genet. 2018 Mar 26;14(3):e1007252. doi: 10.1371/journal.pgen.1007252 (PMC5886687; doi:10.1371/journal.pgen.1007252)
Supplement: S1 Text — (PDF) [file pgen.1007252.s010.pdf]

Supporting Methods

S3 Fig. Premature EGA is determined by the promoter, rather than the 3'UTR.

For RNAi, #1489 animals expressing the EGA reporter (with an unregulated *tbb-2* 3'UTR) were fed with bacteria expressing dsRNAs [1] (targeting *csr-1* from the Ahringer library, or an "empty" vector as a negative control) from L1 stage until adulthood at 25 °C. Adult animals were then examined for expression of the EGA reporter in germ cells.

S4 Fig. PSer5 and PSer2 epitopes in CSR-1^{SIN} oocyte nuclei are Pol II-dependent.

For RNAi, CSR-1^{SIN} animals (#1900) were fed with bacteria expressing dsRNAs [1] (targeting *ama-1* from the Ahringer library, or an "empty" vector as a negative control) from L3 stage until adulthood at 25 °C. The gonads of these animals were then immunostained against PSer5 and PSer2 of Pol II CTD as described in the main manuscript.

S5 Fig. Transcription factor binding site analysis.

For "not expressed in soma early embryonic genes" (see Fig 4, n=250), we tested if transcription factor (TF) binding in the promoter could explain why a subset of those genes had a tendency to go up in *drh-3(rrr2)* mutants. Using a compendium of TF weight matrices (n=220) [2], we scanned the promoters of the *C. elegans* genes (using stretches from 1500 bp upstream to 500 bp downstream of the transcription start site), looking for possible enrichment of TF binding sites. Specifically, we tested, for each TF, if genes containing the corresponding binding site showed change in *drh-3(rrr2)* mutants, compared to genes that did not contain the binding site. With multiple testing correction, a significant hit should show a directed p-value of greater than -log10 (0.05/220)=3.64 (see S5 Fig, gray horizontal line).

S6 Fig. CDL-1 depletion does not cause EGA reporter expression in germ cells.

For RNAi, #1284 animals expressing the EGA reporter were fed with bacteria expressing dsRNAs [1] (targeting *cdl-1* from the Ahringer library, or an "empty" vector as a negative control) from L1 stage until adulthood at 25°C. Adult animals were then examined for expression of the EGA reporter in germ cells.

1. Timmons L, Fire A. Specific interference by ingested dsRNA. Nature. 1998;395(6705):854. doi: 10.1038/27579. PubMed PMID: 9804418.

2. Narasimhan K, Lambert SA, Yang AW, Riddell J, Mnaimneh S, Zheng H, et al. Mapping and analysis of Caenorhabditis elegans transcription factor sequence specificities. eLife. 2015;4. doi: 10.7554/eLife.06967. PubMed PMID: 25905672; PubMed Central PMCID: PMC4434323.
